# School Satisfaction and Self-Efficacy in Adolescents and Intention to Drop Out of School

**DOI:** 10.3390/ijerph21010111

**Published:** 2024-01-18

**Authors:** Maria Luisa Pedditzi

**Affiliations:** Department of Education, Psychology and Philosophy, University of Cagliari, 09123 Cagliari, Italy; pedditzi@unica.it; Tel.: +39-0706757519

**Keywords:** school dropping out, satisfaction in life, self-efficacy, adolescence, school dropout prevention

## Abstract

School dropout is a risky behaviour that is a threat to well-being in adolescence. This study aimed to analyse school satisfaction and self-efficacy in school activities in a sample of adolescents attending secondary school in an Italian region at high risk of school dropout. The objective was to investigate whether differences exist among students on the basis of school dropout intention, gender, and career choices. Another aim was to identify, among the students’ satisfaction variables, the main psychosocial predictors of dropout intention. Students (N = 1340) attending secondary schools in Sardinia completed Soresi’s questionnaires on life satisfaction and self-efficacy. The data were analysed with a multivariate analysis of variance and logistic regression analysis. The results indicated that students intending to drop out of school scored lower on satisfaction with perceived support and satisfaction with peer and teacher relationships than their peers not at risk of dropping out. The logistic regression analysis showed that the most significant predictors of dropout intention were academic performance, satisfaction with the school experience, satisfaction in the relationships with teachers and with family members, and satisfaction with perceived support (26.9% of model variance). The results of this research thus indicate which areas could be addressed through prevention to improve well-being conditions in education.

## 1. Introduction

School dropout involves all the students who leave school without graduating [1,2]. School dropout is a dynamic and cumulative process [3] and is the result of a multifactorial process, which highlights the complex interaction among several variables [2,4,5]. Although different taxonomies have been considered to discriminate the determinants of early school leaving, there is a general agreement in the literature that dropping out is the combined and complex effect of several factors [6].

Many literature reviews [2,4,7] have analysed which of these factors can expose adolescents more to the risk of dropping out of school, and they identified individual, institutional, socio-economic, peer relational, family, and school factors as determinants of early school leaving. The most important individual factors [5] are academic achievement (failure, grade repetition, and academic performance), behaviour (drug and alcohol use, criminality, quality of peer relationships), attitudes (beliefs, goals, values, and self-knowledge), socio-economic background, and health factors (physical and mental health). Some of these factors are considered stronger than others: academic performance, absenteeism, socio-cultural and economic status, family structure, peer relationships, risk behaviours, and mental health have a greater impact [8]. Among the psychological variables of students related to dropping out of school were considered levels of anxiety, depression, stress, and general mental health [4].

The risk of dropping out of school is therefore a complex and global phenomenon that involves multiple factors [9]. Hence, some studies have focused on the intention to drop out of school, using items such as: “I often consider dropping out of school” and “I intend to drop out of school” [10] or similar questions [11]. Other studies have instead focused on the intention to persist in studies [12], taking into account students’ motivation.

Most studies on dropout intention have concentrated on university students [13], and studies on school dropouts in secondary school have tended to focus mainly on the topic of school motivation [14] and the topic of adolescence and risky behaviour [15]. There is a gap in the literature regarding the predictors of the intention to drop out of secondary school, especially considering the school satisfaction variables in the first year of secondary school. This gap probably relates to the fact that the declared intention to drop out is generally associated with past research on older students who therefore found themselves attending high school or university. The intention to drop out of school should instead be considered an important risk indicator starting from the first year of secondary school to be analysed in relation to school satisfaction and study performance. Dropping out is in fact often preceded by a period of psychological distress, during which the student may express dissatisfaction and their intention to leave school [1].

Many students express the intention to drop out of school in adolescence, in a period of transition from secondary school to high school, particularly in the Italian school system. In Italy, school dropout from upper secondary school is mainly a problem in the south. The territorial gaps reflect differences in socio-economic environments, family re-sources, school infrastructure, and, in particular, relating to employment opportunities [16]. Among southern Italian regions, Sardinia ranks first in terms of school dropouts [1,17]. Data from 2019 to 2020 show that 23% of Sardinian 18- to 24-year-olds do not have a diploma. Although below the Italian target of no more than 16%, the school dropout rate in Sardinia is still distant from the EU 2020 benchmark of 10% [1,18,19]. A further aspect that characterises Italian schools is that the choice of upper secondary education (lyceum, technical institute, or institute for specific professions) takes place at age 13, an age at which students are often not yet mature and risk experiencing the school transition with discomfort. Career track choice is left to the students and their families since teachers’ recommendations at the end of Grade 8 are not binding.

Secondary education in Italy is divided into two stages: (1) lower secondary school or middle school (ages 11–13) and (2) upper secondary school or high school (ages 14–19). The two school systems are differently organised, and this transition often leads to destabilisation in the student’s life [20]. From this perspective, school dissatisfaction might be considered as comprising the first stage of a possible pathway, during which a concurrent impact of risk factors might expose students to the risk of dropping out [1].

### 1.1. Subjective Satisfaction

Subjective satisfaction regarding life quality is a psychological topic that has been widely debated in the literature [21,22] within studies on well-being and mental health [23,24]. Life satisfaction is an important construct defined as a cognitive component of subjective well-being, as a conscious judgment of an individual’s own life [25]. It is the subjectively perceived quality of life in multiple life domains, and it is therefore a multi-dimensional construct [26]. Among the life domains considered most relevant in adolescence, Huebner [27] indicated school experience, relationships with family and friends, the living environment, and the relationship with oneself. Research on adolescents has revealed that satisfaction is a significant predictor of positive outcomes in a variety of life areas [10], such as school experiences, classmate and family relations, perceived support, autonomous decision-making, and life conditions [28].

Research based on feedback provided by adolescents has shown that academic success can influence school satisfaction and well-being [19,29]. School satisfaction is also particularly influenced by factors such as encouragement and social support [8,29]. Positive teacher–student relationships can influence student satisfaction with the school experience [19,29,30]. Students who are not consistent in their studies seem to harbour a less positive perception of their relationships with their teachers than other students [31,32]. Studies on satisfaction in relationships with peers indicate that the satisfaction expressed by adolescents depends on the quality of relationships in the classroom and varies by groups [33], age [34], and gender [33,35,36].

Studies on the school satisfaction of adolescents have tended to highlight satisfaction values that tend to decrease in the transition from childhood to adolescence [34], which are generally lower among girls than boys [35,36]. These findings have mostly been attributed to the changes and distress of adolescence. However, other studies have highlighted higher satisfaction scores for girls than boys in supportive relationships [33]. Other studies yielded no significant gender differences per life satisfaction [27]. Inconsistencies between the studies have been defined due to different ages of the study populations and different contexts. Studies conducted by Soresi and Nota [37] have highlighted differences in life satisfaction by gender. Boys seem to be more satisfied than girls in their relationships with classmates and in their current life; conversely, girls seem to be more satisfied in the support received. These studies have mainly focused on students attending the last years of upper secondary school, without specifically focusing on pre-adolescents at risk of dropping out of school [19,38].

### 1.2. Students’ Self-Efficacy

Self-efficacy is a person’s perception of their ability to adequately perform predetermined tasks [39], and the belief in one’s ability to successfully perform designated activities even in the face of challenges [38,39]. Academic self-efficacy shows a student’s level of confidence or belief that they can successfully accomplish educational assignments and tasks, reducing the likelihood of dropping out [40,41]. Students’ self-efficacy beliefs have a substantial impact on the academic performance and on future careers [42] and in predicting their career choices [18,42]. Social cognitive theory [39] focuses on the sources that may contribute to self-efficacy. Butz and Usher [43] found that “mastery experience” and social persuasion were the most frequently reported sources. In high school, social relations during school can be considered important factors in the development of self-efficacy and school satisfaction. Moreover, the support of significant others is extremely important; it is a protective factor for school adjustment during the transition from middle to high school [44]. Some studies have analysed the relationship between teacher support and self-efficacy beliefs [45], underlining that teachers and parents influence optimism, inspire self-efficacy beliefs, and shape students’ positive attitudes toward their academic future [45]. Research, however, has suggested that there are sex, racial, and ethnic disparities in academic self-efficacy [46,47].

In general, it seems that ethnic and racial minorities appear to have lower levels of academic self-efficacy [48]. Peguero and Shaffer [49] underlined how the intersection of sex, race, and ethnicity matters in the link between academic self-efficacy and the likelihood of dropping out.

Some studies [37,38] highlighted gender differences in self-efficacy associated with decision-making in school activities. Boys generally tend to obtain higher scores than girls. However, this seems to be partly associated with cultural factors with respect to the expression of one’s abilities per self-report scores [19,38].

Given the possible socio-cultural differences of students, research was carried out to verify whether in contexts with a high risk of dropping out of school, such as Sardinia, gender differences persist in self-efficacy and in satisfaction between students at risk of dropping out and students not at risk. The present research therefore specifically aimed at investigating whether there are differences among preadolescents in terms of dropout risk and gender.

The previous studies conducted in upper secondary schools in Sardinia analysed students’ school satisfaction and self-efficacy but did not consider students’ intentions to drop out in the first year of secondary school [19,38]. We also aimed to verify, among the main psychosocial components of student satisfaction, the most significant predictors of school dropout intention.

Considering the purpose of this research, the following research questions were developed:

(1) Within the students of the sample examined, are there differences in school satisfaction and self-efficacy by gender and by intention to drop out of school?

(2) Within the students of the sample examined, are there differences in school satisfaction and self-efficacy by post-graduation choice intention and by intention to drop out?

(3) Within the students of the sample examined, which are the most significant predictors of school dropout intention among the main psychosocial components of school satisfaction considered?

## 2. Method

This study was cross-sectional and descriptive, involving 1340 students, balanced by gender, who were attending upper secondary schools in Sardinia (Italy). Data collection took place during school hours. The ethics committee of the Department of Pedagogy, Psychology, and Philosophy at the University of Cagliari approved the research (Prot. 10/07/2018, No. 25). Participants completed the questionnaire individually, and the response rate to the questionnaire was 92%.

### 2.1. Participants

The participants were 1340 students attending the first year of upper secondary school in Sardinia. More than half (59.6%) were boys (N = 798), and 40.4% were girls (N = 542; M age = 14.6, SD = 1.1). A little more than one third (36.3%, n = 486) were attending high schools, 34% (n = 456) were attending technical institutes, and 29.7% (n = 398) were attending institutes for specific professions.

The schools were selected through convenience sampling on the basis of their willingness to join the research project in a region of southern Italy that is particularly at risk for early school dropout. About one quarter (25.4%, n = 341) of the students had repeated at least one school year.

More than half of the students said they had thought about dropping out of school (57.9%, n = 776), and 42.1% (n = 564) were students progressing with their studies who declared that they had never thought of leaving school.

Regarding intentions about future choices, 54.1% (n = 725) of the students intended to work immediately after high school graduation, 39% (n = 522) intended to continue their studies at university, and 6.9% (n = 93) said they did not know what to do after graduation.

### 2.2. Instruments

To assess students’ satisfaction in different school and life domains, the My Student Life Questionnaire by Soresi and Nota [37] was used, and specifically the scales:

- School Experience (seven items, e.g., “I am really satisfied with the school I am attending”; α = 0.86);
- Relationships with Classmates (three items, e.g., “I can say that I really talk a lot with my classmates”; α = 0.70);
- Relationships with Family Members (four items, e.g., “With my family I get along just fine”; α = 0.84); and
- Perceived Support (two items, e.g., “In case of need, I know where to find those who can help me”; α = 0.79).

The respondents were instructed to rate themselves on each item using a 5-point Likert scale (1 = not at all satisfied, 5 = very satisfied). The psychometric requirements of the instrument are given in Soresi and Nota [19].

To measure satisfaction with teacher–student relationships, we adopted a scale taken from the Programme for International Student Assessment, designed by the Organisation for Economic Co-operation and Development [50], and we obtained comparable data regarding students’ success levels in 32 different countries.

This scale has been recently used [51,52] to assess teacher–student relationships in secondary school, and the results have shown that the internal coherence of the items is equal to α = 0.78. The items in this scale include the following statements: “I get along well with most of the teachers”; “Most of my teachers really listen to what I have to say”; “If I need extra help, I will receive it from my teachers”; and “Most of my teachers treat me fairly” (α = 0.87, comparative fit index (CFI) = 0.967, Tucker–Lewis Index (TLI) = 0.947, root mean square error of approximation (RMSEA) = 0.08).

To assess students’ self-efficacy in school activities and choices, we used the Clipper Questionnaire’s item, “How Much Confidence do I Have in Myself?” by Soresi and Nota [37]. For this study, we decided to focus on the Students’ Confidence in Their Ability to Carry Out Tasks and School Activities subscale.

We considered it to be the most adequate scale to evaluate the impact that a new school context may have on students. The four items in this scale include the following statements: “I think I can learn almost everything”; “I think I can do many things”; “If the others got to know me well, they would say that I can do almost anything”; and “I am so confident in my abilities that sometimes I like dealing with difficult things”. The one-factor solution was replicated in our sample (Cronbach’s α = 0.80, CFI = 0.980, TLI = 0.941, RMSEA = 0.08). Each student answered the questions on a 5-point Likert scale (1 = not at all satisfied, 5 = extremely satisfied).

We also used a sociographical form to analyse variables such as gender, age, academic performance in the first quarter, past and future schooling intentions, and regularity in studies. Specifically, participants were asked if they had ever had the intention to drop out of school. This question was followed by a dichotomous response pattern (1 = no, 2 = yes) as in other previous studies [11].

### 2.3. Data Analysis

To answer the first research question, we conducted a factorial multivariate analysis of variance (MANOVA) to assess whether some independent grouping variables (in our case, at first, gender and the risk of school dropout) explain a statistically significant amount of variance in the questionnaire’s scales. The gender variable included two factors (boys and girls), and “risk of school dropping out” included two factors (yes and no). All subgroups were balanced.

To answer the second research question, we applied again the MANOVA using other independent variables: type of career choice (two factors: university vs. work) and risk of dropping out of school (two factors: yes, no).

Finally, a linear logistic regression analysis was performed, using all satisfaction variables to identify the most significant predictors of school dropout intention (third research objective). Dropout intention was used as a dichotomous dependent variable (yes, no), and all the other school satisfaction variables and academic performance were used as predictors. The significance level for all statistical analyses was *p* ≤ 0.05.

## 3. Results

The results of this research are reported below. Section 3.1.1 shows the results of the first MANOVA as an answer to the first research question; Section 3.1.2 reports the results of the second MANOVA as an answer to the second research question. Finally, in Section 3.2, the results of the logistic regression analysis are reported as an answer to the third research question.

### 3.1. MANOVA Results

#### 3.1.1. Risk of School Dropout and Gender

The MANOVA highlighted significant differences in satisfaction with quality of life and self-efficacy in school activities by gender (Wilks’s λ = 0.961, F = 10.716, df = 5, sig = 0.0001, *p* < 0.05) and by school dropout intention (Wilks’s λ = 0.958, F = 11.579, df = 5, sig = 0.0001, *p* < 0.05), without effects of a Gender × Risk of Dropping Out interaction. Students who intended to drop out scored lower in school satisfaction (n = 776; M = 3.512, SD = 0.810) than their peers who did not intend to drop out (n = 564; M = 3.786, SD = 0.815). They also scored lower on “satisfaction with the support received” (n = 776; M = 3.646, SD = 1.06) than regular students (n = 564; M = 3.88, SD = 1.07) and in “satisfaction with peers” (n = 776; M = 3.413, SD = 0.780 vs. n = 564; M = 3.486, SD = 0.890). On the Positive Relationship With Teachers scale, students at risk of dropping out of school also obtained the lowest scores (n = 776; M = 2.953, SD = 0.937 vs. n = 564; M = 3.295, SD = 0.942). In “satisfaction with peer relationships”, girls (n = 542; M = 3.330, SD = 0.908) scored lower than boys (n = 798; M = 3.521, SD = 0.761). They also reported lower scores in “self-efficacy in choices” (n = 542; M = 13.383, SD = 3.56) than boys (n = 798; M = 14.280, SD = 3.20).

#### 3.1.2. Risk of School Dropout and Career Choices

The MANOVA showed significant differences in satisfaction with quality of life and self-efficacy in school activities on school dropout intention (Wilks’s λ = 0.964, F = 9.301, df = 5, sig = 0.0001, *p* < 0.05) and by career choice intention (Wilks’s λ = 0.984, F = 4.104, df = 5, sig = 0.001, *p* < 0.05), with interaction effects between these two variables (Wilks’s λ = 0.991, F = 2.279, df = 5, sig = 0.045, *p* < 0.05). Students at risk of school dropout (n = 723) scored lower on school satisfaction (M = 3.529, SD = 0.799) than their peers not at risk (M = 3.795, SD = 0.808). They also scored lower on satisfaction with support received (M = 3.663, SD = 1.06 vs. M = 3.874, SD = 1.07) and satisfaction with teachers (M = 2.978, SD = 0.903 vs. M = 3.334, SD = 0.916). Students intending to choose a career path immediately after graduation (n = 725) reported lower scores in “school satisfaction” (M = 3.546, SD = 0.828) than students intending to enrol in university (n = 522; M = 3.772, SD = 0.775). They also scored lower in “satisfaction with the support received” (M = 3.675, SD = 1.05 vs. M = 3.858, SD = 1.08), in “satisfaction with family members” (M = 3.409, SD = 0.851 vs. M = 3.565, SD = 0.827), and in “satisfaction with teachers” (M = 3.01, SD = 0.928 vs. M = 3.279, SD = 0.900). The interaction effect between school dropout intention and career path choice affected only school satisfaction (F = 2.279, df = 5, sig = 0.045, *p* < 0.05).

### 3.2. Predictors of Dropout Intention

Our third research question aimed to determine whether satisfaction in different areas of school life and academic performance significantly predicted the students’ dropout intention. Table 1 represents the results of a logistic linear regression analysis carried out using dropout intention as the categorical dependent variable (yes, no) and the school satisfaction variables and academic performance as predictors.

When Table 1 is examined, one can see that dissatisfaction with the school experience (Wald = 13.437, *p* < 0.05), dissatisfaction with teachers (Wald = 13.395, *p* < 0.05), dissatisfaction with family members (Wald = 8.706, *p* < 0.05), dissatisfaction with the support received (Wald = 6.808, *p* < 0.05), and low academic achievement (Wald = 82.620, *p* < 0.001) were significantly predictive of school dropout intention. Students who were school dissatisfied in different areas of school life, that is, school experience (OR = 0.646, 95% CI = 0.511; 0.816), relationships with teachers (OR = 0.695, 95% CI = 0.572; 0.844), relationships with family members (OR = 1.139, 95% CI = 0.903; 1.425), support received (OR = 0.814, 95% CI = 0.697; 0.950), and academic performance (OR = 0.521, 95% CI = 0.452; 0.599) were significantly more likely to harbour a school dropout intention than those who were most satisfied.

The Negelkerke R^2^ and Hosmer–Lemeshow tests were also computed. Negelkerke R^2^ values of the independent variables related to predicting the variance indicated for the dependent variable. According to Negelkerke R^2^, providing an opportunity to predict the variance, all satisfaction areas (excluding satisfaction with classmates) explained 26.9% of the dependent variable.

The result of the Hosmer–Lemeshow test, which evaluates the goodness of fit of the model as a whole, did not give a significant value, χ^2^(8) = 11.230, *p* > 0.05. The fact that this value was not significant indicates that the model had an acceptable fit and that the data fit of the model was at a sufficient level.

Consequently, the findings obtained from this study indicate that the students’ school satisfaction and their academic performance had positive influences against school dropout.

## 4. Discussion

The results of this research highlight the presence of low levels of satisfaction with quality of life and self-efficacy in students at risk of dropping out of school. Specifically, students who intended to drop out of school reported low levels of satisfaction with the support they received and low satisfaction in their relationships with teachers and schoolmates compared with classmates not at risk of dropping out. Their levels of self-efficacy in school activities and school satisfaction were also low and oriented toward intentions of choice for a non-academic future. Their choice to undertake non-academic postgraduate paths was associated with their dissatisfaction with relationships with teachers and their dissatisfaction with the support they received. The results of this research confirm the data present in the literature on individual and educational risk factors for school dropout [6], which mainly refer to variables such as academic failure, grade repetition, and academic performance.

The results relating to the life satisfaction and self-efficacy of students at risk of dropping out describe the possible choices for the future of young people at risk of dropping out in relation to their intention to drop out. The results concerning decision-making self-efficacy and satisfaction also confirm the previous studies conducted in Italy [19,38] on the differences between girls and boys attending secondary school. Compared to previous studies in the literature, these results focused on students in the first year of upper secondary school. Previous studies conducted in Sardinia had focused in particular on older students in relation to post-diploma choices [19,38].

As in previous research, it was therefore confirmed that the girls in our study also continued to report lower scores than boys in self-efficacy regarding the ability to carry out various activities in the future, which could reflect, on the part of girls, a greater caution in expressing positive expectations about their professional future than the boys [19,38,53]. It would be interesting to investigate in future studies how the female students in the considered context represent their skills with respect to their scholastic and professional futures in order to grasp the possible limits they perceive regarding their future professional placements.

In our sample of secondary school students, as also evidenced by the results of our logistic regression analysis, students’ satisfaction and academic achievement were predictors of dropout intention. Almost all variables of student satisfaction (satisfaction with the school experience, with student–teacher relationships, with family members, and with support received) were therefore able to predict school dropout intention. Satisfaction in relationships with classmates is probably not among the predictors of the intention to drop out of school due to the possible variety of class contexts and groups within which students find themselves living their relationships at school [33]. Although academic performance has been considered one of the main predictors of school dropout [8], school satisfaction referring to psychosocial variables specific to the school context (relationships with teachers, classmates, support received, school experience, and family) had not yet been considered as predictors of the intention to drop out from the first year of upper secondary school in a sample at risk of school dropout in a place like Sardinia.

Because this study was conducted with a specific convenience sample, we note that these results must be considered in specific relation to our particular research sample without any pretensions to generalise our findings to the category of Italian students in general. Another limitation is its shared method variance because all measures were filled out by the same participants. Furthermore, in future research, the intention to drop out of school could be evaluated more widely by employing a standardised test. This method would enable a more comprehensive investigation of this variable, moving beyond its binary classification.

## 5. Conclusions

The results of this research underline the importance of school satisfaction and self-efficacy as subjective variables in predicting school discomfort and the risk of dropping out. Timely interventions in school contexts to improve the levels of these variables would prove useful in preventing school dropouts. Further interventions might focus on the psychosocial dimension of adolescents’ satisfaction in their relationships with their parents, teachers, and classmates and on their perceptions of the support received.

In recent discussions of school well-being, the WHO [54] recommended promoting the prevention of student distress at school, and recent research has found empirical evidence demonstrating that students’ satisfaction and life skills could improve perceived well-being in adolescence [38,55,56].

It is also worth mentioning the need to create both psychological counselling courses based on the specific moment of transition in which students find themselves and intervention programs to counteract risk factors and encourage protective ones [57,58,59], in particular in communities where the risk of dropping out of school is high. This should encourage teachers and educators to reflect on the importance of facilitating early educational initiatives aimed at preventing psychosocial dissatisfaction and distress at school.

The interventions could be aimed at improving both students’ levels of satisfaction with school and their relationships with teachers, especially for students at risk of dropping out. As previously found in studies on early school leaving [1,19,33], the socio-relational component and a good school climate can help promote students’ well-being and their academic results. This is interesting from the point of view of the possible interventions that could be implemented in schools to promote self-efficacy in girls regarding their choices for the future. A future investigation could more generally assess students’ future choice intentions, like in the Sardinian students we interviewed.

Only a few students in the sample reported university as an alternative future choice, whereas a very high percentage of students declared that they wanted to look for a job immediately after graduation.

In a financially poor context with high youth unemployment such as Sardinia [16,20], clearly declaring with certainty that you want to enrol in university is probably not common. At the time of data collection and in the presence of scenarios for the future increasingly characterised by insecurity, especially in the presence of problems related to early school dropout, this study underlines the need to prepare young people to live with future challenges.

## Figures and Tables

**Table 1:** Summary of logistic regression on dropout intention.

Scales	B	SE	Wald	df	sig	EXP(B)	CI for 95.0% EXP(B)	
							Lower	Upper
Satisfaction with the school experience	−0.437	0.119	13.473	1	0.000	0.646	0.511	0.816
Satisfaction in relationships with teacher	−0.364	0.099	13.395	1	0.000	0.695	0.572	0.844
Satisfaction in relationships with classmates	−0.185	0.103	3.188	1	0.074	1.203	0.982	1.473
Satisfaction in relationships with family members	−0.292	0.099	8.706	1	0.003	1.139	0.903	1.425
Satisfaction with support received	−0.206	0.079	6.808	1	0.009	0.814	0.697	0.950
Academic achievement	−0.653	0.072	82.620	1	0.000	0.521	0.452	0.599
Constant	2.579	0.455	36.763	1	0.000	15.790		

Negelkerke R^2^ = 0.269; Hosmer–Lemeshow test χ^2^(8) = 11.230, *p* = 0.34; *p* < 0.001.

## Data Availability

Research data are available by writing to the author.
